# Dealing with Chronic Illness: Experiences of Iranian Families of Persons with Multiple Sclerosis—A Qualitative Study

**DOI:** 10.1155/2017/9243161

**Published:** 2017-09-07

**Authors:** Hossein Ebrahimi, Hadi Hasankhani, Hossein Namdar, Esmail Khodadadi, Marjaneh Fooladi

**Affiliations:** ^1^Psychiatric Nursing Department, Faculty of Nursing and Midwifery, Tabriz University of Medical Sciences, Tabriz, Iran; ^2^Qualitative Studies Center, Tabriz University of Medical Sciences, Tabriz, Iran; ^3^Research Department, Faculty of Nursing and Midwifery, Tabriz University of Medical Sciences, Tabriz, Iran; ^4^Faculty of Nursing and Midwifery, Tabriz University of Medical Sciences, Tabriz, Iran; ^5^University of Jordan, Amman, Jordan; ^6^World Wide Nursing Service Network (WWNSN), PLLC, El Paso, TX, USA

## Abstract

**Background:**

Today family members are providing care and support to each other during illness. In particular, in chronic illness, such as multiple sclerosis, the families are more involved in caring for and supporting their patients, so they use several strategies to cope with this situation. The purpose of this study was to explore the coping strategies in family caregivers of persons with multiple sclerosis in Iran.

**Methods:**

This is a qualitative study that was conducted through 18 family caregivers of persons with multiple sclerosis. A purposeful sampling method was used. Data were collected through semistructured and in-depth interviews conducted in Multiple Sclerosis Society and hospitals of Tabriz in Iran. The collected data was analyzed according to qualitative content analysis.

**Results:**

Five main categories were elicited from interviews: “using spirituality,” “living with hope,” “experiencing persistence and stability,” “seeking support,” and “seeking alternative treatments.”* Conclusion*. The study findings can help to inform the support given to families to help them cope with the effects of caring for someone with multiple sclerosis. Health system managers and professionals by using these results are able to support patients and their families appropriately in order to improve their quality of life and alleviate the complications of disease.

## 1. Introduction

Multiple sclerosis (MS) is a chronic neurological illness, with no known cure, and is manifested in various types, with the primary progressive form being one of the most debilitating forms [1]. The central nervous system (CNS) involving the brain and spinal cord in young people with MS is aggressively attacked with a rapid degeneration of the myelin sheath of nerves in most cases, leading to the erratic periods of recovery and recurrence [2]. The MS etiology is unknown but hypothesized as a genetic condition influenced by environmental factors [3]. The lesions in MS have an effect on patients' physical strength, sensation, cognition, vision, and coordination [4], requiring long-term care, especially among the younger people with a more progressive form over many years [5]. Thus, family caregivers become a significant part of care for their family members and their wellbeing under severe psychological stress is a major concern [6, 7].

According to the MS International Federation global estimates, there are 2.3 million people diagnosed with MS [8], with nearly 400,000 in the United States [5]. According to the Iranian Neurological Society, the number of persons with MS is on the rise with 51.9/100,000 in 2009 to 73/100,000 in 2011 [9]. Limited statistical data on the MS prevalence shows an estimated 60 to 70 thousands of cases in Iran [10].

As an unpredictable chronic condition with unknown prognosis, MS is experienced differently among patients [11], and family caregivers assuming responsibility for the constant care often experience emotional distress and sadness with role modifications [12, 13]. On the other hand, patients, their spouses, and other family members face numerous economic and social challenges such as losing a job, reduced income, changes in social status, and caregiver exhaustion [14, 15].

As the MS disease progresses, patients need more care and family caregivers carry a heavier responsibility for their care [16]. Studies on chronic illnesses have reported that family caregivers provide 80 percent of lifetime care for their patients [17]. Coping with long-term care of a chronic condition such as MS demands family support for caregivers [14] and most studies focus on the disease process or patient and ignore the needs of family caregivers for support [18].

Study of persons with MS and how their family adapted to a chronic condition found that most families managed by using coping strategies in search of solutions to problems rather than wishful thinking [19]. Other studies have shown that economic hardship has a significant effect on the welfare and mental status of persons with MS and their families, where extra medical expenses become a stressor, especially among those depending on disability pension [20–22]. Economic despair often originates from the prohibiting aspect of MS for continued employment, hospitalization, healthcare services, and daily medication expenses [23, 24]. It is essential for the healthcare system to provide effective services based on needs of persons with MS and family support [25].

In Iran, MS is a relatively rare and unknown condition and majority of Iranians do not know about associated compilations or what type of care is needed to manage MS [26]. This qualitative study was conducted to explore and describe family caregivers' understanding, experiences, and coping strategies when caring for persons with MS. Researchers examined family and social perspectives in Iran and aimed to identify areas that need improvement.

## 2. Methods

### 2.1. Study Design

Using a qualitative and conventional content analysis research method, 18 Iranian family caregivers were approached to explore their experiences when caring for persons with multiple sclerosis [27]. Purposive sampling was used to select suitable participants with rich information as a caregiver. Theoretical sampling helped refine and improve concepts and categories expressed by participants who had significant experiences and continued until data saturation was reached. Verbal, visual, and written data were analyzed using a qualitative content analysis method [28].

### 2.2. Participants

The study participants were 14 female and 4 male family caregivers of persons with MS and consisted of closed relatives, husbands, wives, and parents. As presented below, the family caregivers' age range was between 26 and 54 years and the mean duration of caregiving history was 3.6 years (Table 1).

### 2.3. Recruitment

To recruit participants, flyers were distributed at the local clinics, hospitals, and Multiple Sclerosis Society, inviting family caregivers to join the study. The inclusion criteria consisted of caregiver who consented to participate, have at least six months of caregiving, and are closely related to the persons with MS. Eighteen family caregivers were eligible to participate. The study site included facilities at Multiple Sclerosis Society, Internal Medicine and Neurology Hospital wards in Tabriz, Iran, during May to December 2015.

### 2.4. Data Collection

Data were first collected through unstructured in-depth interviews using general questions on personal experiences as a caregiver of persons with MS and continued with semistructured questions asking “how do you meet the patient's needs?” and “how do you manage the MS condition on daily basis?” Field notes were a part of data collection as participants shared their experiences. Recorded interviews lasted between 45 and 60 minutes and later transcribed.

### 2.5. Data Analysis

Conventional content analysis was used to analyze data [27]. Interviews were transcribed verbatim and analyzed concurrently during the data collection process. Verbatim data were analyzed line by line to extract the primary codes and later classified according to their similarities and differences. Concepts and subcategories were extracted and defined and coded to find the main categories. After using the MAXQDA software 10.0 [29], data were organized and analyzed again to find the five main categories and 18 subcategories (Table 2).

### 2.6. Trustworthiness of the Study

To ensure research rigor, group debates and discussions among the researchers continued until consensus was reached. The primary analysis and results were shared with participants to verify accuracy and validate coding congruity of personal experiences. Data credibility was established through long-term engagement with each participant. Partial review of literature at the onset helped reduce researchers' bias and increase dependability of data gathering and analysis processes. Confirmability was achieved by cautiously recording and reporting the study steps toward decision-making and further research. The highest degree of diversity in participant selection was intended to amplify transferability [30].

### 2.7. Ethical Consideration

This study was approved by the Regional Committee of Medical Research Ethic at Tabriz University of Medical Sciences. An agreement was obtained from the Multiple Sclerosis Society and hospital managers to recruit family caregivers of persons with MS. Participants were informed of the study objectives prior to signing an informed consent outlining their voluntary participation and regard for confidentiality of personal data.

## 3. Results

Five main categories were elicited from interviews including: “using spirituality,” “living with hope,” “experiencing persistence and stability,” “seeking support,” and "seeking alternative treatments." Table 2 provides a list of 5 main categories and 18 subcategories.

### 3.1. Using Spirituality

The strength from spiritual beliefs was evident in the accounts of participants' experiences in search of peace by relying on God and praying. They trusted God when facing difficulties and experiencing helplessness. Their prayer helped them find peace, tolerate hardship, and have their loved one regain wellness soon.

#### 3.1.1. Trust in God

Participants' faith and trust in God was instrumental for their emotional survival as they sought God's help in difficult situations.

I asked for healing from God and only trusted him. The thought of losing my young husband was really disturbing to me. In loneliness I spoke to God in my prayers …. (P18)

#### 3.1.2. Sincere Worship

When someone believes in and confides in God, he or she feels mentally relieved and relaxed. Participants reported that they felt a special relaxation after worshiping God and regained strength to tolerate any problem more easily.

When you need relatives' support, they leave you. Then, you would feel alone and depressed. During these moments, worshiping God brings you hope. When I pray to God I feel brave and reassured in my heart. I seek God's mercy in my prayers…. (P4)

#### 3.1.3. Faith in God

Faith in God is a deep belief guiding a person and giving him or her encouragement. Patients' families lived a religious life with faith and managed their lives in God's mercy.

I think God helped me when he was very ill and gave me patience. My faith in God and my religious beliefs did not let me surrender to problems in difficult times or when I was too tired. My faith encouraged me to take care of my husband …. (P8)

#### 3.1.4. Belief in Destiny

The Islamic culture consists of a belief in destiny, where a higher power such as God's will controls the outcome and a true believer must surrender to God's will. The family caregivers were Muslims and believed in destiny designed by God and were ready to accept it.

When I found out that my wife suffers from MS, first I was very sad, but soon I began to focus on hope rather than sadness and reminded myself of God's will. Whatever God as the ultimate power wants for my destiny, I accept it. I surrender to God's will and only ask for patience …. (P10)

#### 3.1.5. Relying on Religious Values

Religious beliefs are sacred for Muslims and used as a guide for their thoughts and actions in life. Accordingly, they seek intercession and healing for the sick as a part of their religious beliefs.

Since I am a Sayyed (descendants of the prophet), it is my religious duty to be a caregiver for my family. I took care of her from the beginning until now and I feel satisfied. I vowed that when she recovers, I will bring her to Imam Hussein shrine in Karbala pilgrimage …. (P3)

### 3.2. Living with Hope

Being hopeful for a successful treatment and recovery eased participants' anxiety and optimistic mindset toward future strengthened patient's morale when family caregivers showed less tension and stress. Participants found living with hope played an overall key role.

#### 3.2.1. Hopefulness

Participants induced hope in patients to strengthen their morale. They downplayed the progressive nature of MS, even in critical times by focusing on hope. The family caregivers suggested that being hopeful about future made them more resilient and reduced exhaustion.

Many times I felt stress and exhaustion, but reminded myself to be hopeful for my daughter. I said to myself that I should be strong during difficulties and remain besides her. At times when she asked me if she would ever get better, I would tell her sure! You will go back to your house on your own two feet and live a nice life with your husband and children just like before…. (P2)

#### 3.2.2. Optimism

Optimism fostered hope and a desired feeling that led to self-confidence and hard work. Participants shared that being optimistic conquered despair, revived their hope in life, and made the family atmosphere positive and happy.

Of course we and most patients' families are optimist toward future and always hope that certain cure will be found for our patient to recover completely …. (P13)

### 3.3. Experiencing Persistence and Stability

Despite the ominous prognosis, family caregivers endured the hardship and focused on cure. The progressive effects of MS tested their morale and self-confidence and they continued to persevere with patience against all odds, while managing daily challenges. Family caregivers remained strong and developed resilience when coping with the symptoms of MS.

#### 3.3.1. Maintaining Morale

When family members learned that their loved one had MS, they tried to hide the impact of required long-term care from the patient and maintain high morale as if there was no reason to feel unhappy.

We wanted to keep our feelings hidden…even asked the nurses not to discuss the disease and it complications with the patient to avoid low morale …. (P1)

No, I never got angry in her presence and never said expenses were high because it would affect her morale in a negatively. I was really under pressure, but I said nothing …. (P5)

#### 3.3.2. Self-Reliance

After the diagnosis of MS, family caregivers tried to remain strong and confident that someday their loved one will be well. Through perseverance and self-reliance they began searching for information on how to care for persons with MS and conquer the problems.

When expenses for the disease were high I worked extra hours and spent less. For example previously I bought 3 kilograms of meat but reduced it to 1 kilogram monthly. I had to do that to manage the disease related expenses…. (P6)

#### 3.3.3. Independence in Care

As other family members and healthcare professionals were gradually less available, participants learned how to be self-reliant and care for their loved ones independently. Taking care of their patients without needing others' helped participants gain self-confidence and strengthened their morale.

First I took care of her and did all the injections. I watched and learned how the doctor did it and then injected my wife myself. When I was fairly comfortable, I taught my wife also and now she can take care of herself alone …. (P16)

#### 3.3.4. Managing the Conditions

Upon the MS diagnosis, participants did everything to reduce complications and increase the patient's quality of life. They believed in conquering the disease and limiting its symptoms.

Most families like mine think of certain cure for the disease and continue seeking different medicines. Once we realize that MS has no known cure, we begin to fight the disease with all our power…. (P7)

### 3.4. Seeking Support

Most families encountered difficult challenges and felt alone. Then, they decided to ask for social support through government resources.

#### 3.4.1. Seeking Financial Support

Majority of families suffered from financial setbacks and complained about high cost of medications making them unaffordable in light of low income and employment loss. Some families worked extra hours, borrowed money from relatives and friends, or sought help from charitable organizations or MS Association. Others received financial supports from insurance companies to partially pay for their medical expenses.

The most common and important concern for the family caregivers of MS patients is the high cost of medications putting extra pressures on their finances. They have to work extra hours, seek help from charitable organizations, borrow from relatives and go to government agencies for financial assistance…. (P9).

#### 3.4.2. Seeking Social Support

Persons with MS and their families resist appearing in public or attend family gatherings to avoid people's negative attitudes or being rejected. Therefore, seeking social support from the MS society or MS support groups plays a vital role in strengthening their morale as they adapt to the disease progression.

The MS Association has supportive volunteer personnel and they are devoted to the persons with MS and their families. Some families and philanthropists have made significant financial contributions to the healthcare entities and such kindness gives us hope for the future…. (P17)

#### 3.4.3. Desire for Emotional Support

Some of the patients' relatives continue to accept caregiving responsibility and help the family. This emotional support is very satisfying and they felt hopeful when they saw their friends and relatives near them.

Sometimes relatives and friends come for a visit and that makes us all very happy. Although they do not offer help, their presence shows they care. Close relatives are always supportive in every way. Without their help, I don't know how we could manage our problems. (P11)

### 3.5. Seeking Alternative Treatments

Families remained hopeful and when they realized that prescribed treatment was ineffective, they began doctor shopping according to age, having “enough” experience, or wanting prescription for a “better” medication. As different doctors ‘orders and prescriptions did not show improvement in their loved ones, families partially accepted the reality by losing hope in modern medicine and pursued traditional methods.

#### 3.5.1. Discontinuing Current Treatment

Some families noticed lack of improvement in their patient or did not see a fast recovery as expected and decided to discontinue treatment and stop all medications. The lack of awareness on the nature of MS as a chronic disease and the requirement for prolonged treatment was the major reason for discontinuing treatment. Enthusiasm and expectation for a quick response to treatment subsided and are replaced with impatience, mental anguish, stress, and concerns for the future.

Most families are seeking certain treatment and looking for medications for a rapid recovery. They try all kinds of drugs; some sink into hopelessness and discontinue treatments…. (P16)

#### 3.5.2. Seeking More Effective Medications

Through family support groups medication effects or side effects were shared in search of partial to complete recovery. They used all available information about treatments and compared it with other families' experiences.

We are waiting for a new drug to completely cure MS and to be affordable. We asked our doctor and other patients' families if there have heard of a new drug …. (P11)

#### 3.5.3. Testing New Medication

Often families followed the medical news and as soon as they would hear about a new drug coming to the market, they will buy it and take it without medical consult or demand a prescription from the doctor (in Iran, prescription is not always required). Most families try anything and optimistically look for cure at any cost.

I asked the doctor to change the prescription when he said the new pill can enhance his leg movements and help him walk… he would not need hospitalization as well. (P1)

#### 3.5.4. Resort to Traditional Remedy and Nondrug Treatment

When modern medicine did not produce the expected results, families resorted to nonpharmacological or traditional methods and even voodoo medicine. Families knew that some of the methods were not beneficial, but they tried them anyway as a last chance for healing. Using a network of communication with other families, they would apply acupuncture, bee therapy, leech therapy, herbs, and prayer writing circle with hope for recovery.

Some of us are dismayed with modern medicine producing no result. We are utterly hopeless and do not want to continue with medical treatment and prefer to use non-pharmacological methods such as leech therapy, bee therapy and acupuncture …. (P14)

## 4. Discussion

Researchers aimed to explore the coping strategies that Iranian families of persons with MS used to manage a chronic and debilitating condition. Studies have shown that families with a patient diagnosed with MS try to adapt and adjust living with a chronic illness as a new situation [31, 32]. The findings of this study showed that families of MS patients used various strategies to deal with the condition and its complications.

Theoretically, patients diagnosed with a chronic illness and their families attempt to be optimistic toward regaining good health and willingly adapt to new situations [33]. Stress, coping style, and adaptation to crisis such as chronic illnesses are different for each individual. Various theories have explored adaptability to living with a chronic condition as an important intermediate to achieving mental relaxation in which a person can fulfill his/her exogenous and indigenous needs [34]. Lazarus and Folkman's coping theory was broadly used by participants in this study through active and passive strategies.

Family caregivers in this study tried to achieve calm and control through several mechanisms in order to fulfill their family needs. One of the coping mechanisms was spirituality and use of prayer and worshiping God during difficult times. In Dadkhah et al. (2014), researchers stated that participants sought help from their faith when confronted with the decision on lower limb amputation [35]. Another study reported that religious beliefs supported adaptability to chemical injury [36] or relying on religious beliefs for accepting the breast cancer diagnosis [37]. It is important to consider the sociocultural and religious contexts when examining individuals' adaptability to crisis [38].

Participants in this study used a different coping mechanism regarding MS progression by seeking alternative treatments. Expecting rapid results and efficacy, families tried traditional treatments to recover and prolong life. In other studies, adaptability to living with a chronic condition was associated with the relationship between the patients and family members and the disease severity [39].

The Iranian family caregivers used different coping skills to manage stress [34] by being motivated and strong to uplift their patient's morale with hopeful view of the future. Based on participants' experiences, hope increased morale and eased their stress. In Borneman et al.'s study (2002), the concept of hope for family caregivers was described as an energizing situation providing positive perspective [40]. Several studies have shown that concept of hope has an important role in coping with difficult conditions [36, 37, 41].

When there was a consistent and stable family structure, patients and family caregivers managed the problems more efficiently and as participants in this study struggled with the disease complications they relied on each other to reduce their loved one's suffering. They fostered hope and boosted morale, while caring tirelessly to enhance patients' life quality and mental state. Other studies concur with this finding and suggest that family caregivers continue to care for patients and enhance their life quality by using self-care strategies [42–44].

Adapting and coping with a health crisis required external support as family caregivers in this study discovered. The study participants felt helpless in difficult situation with financial issues, physical and mental exhaustion as they carried the burdens and needed support. Other studies on MS patients and their family caregivers showed that social support played an important role in managing the difficult and complicated conditions [19, 45, 46]. Dadkhah et al. (2014) suggested that social support especially from friends and family played a significant role toward adaptability [35].

Social support brought significant relief for study participants with emotional, informational, and financial assistance. These findings concurred with other studies of family caregivers of patients' with chronic illnesses who received social support in critical periods and helped them better cope with crisis [47, 48]. The support systems included family members, relatives, friends, physicians, and other healthcare providers [48].

One of the most important duties of a family caregiver is taking care of patient's personal hygiene, nutrition, medication administration, and paying close attention to the symptom progression. Spouses, family members, and close friends play an important role in patient's psychological status [49] as the first-line support system. A spouse is the most important person to take care of his/her needs with emotional and physical care in a loving atmosphere [50]. We found the same results suggesting the provision of emotional support for family caregivers as a particularly important aspect of successful coping strategy [51]. Several other studies in Iran also suggested that family caregivers played a vital role in supporting the patients to adapt to a chronic condition [35, 37, 41] by overcoming the challenges in a positive way [16].

## 5. Conclusion

Researchers explored the Iranian family caregivers of persons with MS and examined their coping strategies as they performed caregiving duties and built resilience. We found rich narratives of lived experience, which outlines the Iranian caregivers' experience from a unique perspective. The Iranian family caregivers resolved their problems through self-reliance and faith by maintaining a high morale to help their loved one diagnosed with MS. Relying on spiritual connections, social support, and alternative treatments, family caregivers managed to adapt and provide care. Healthcare providers would benefit from the findings of this study as they communicate, inform, and offer support to the patients and their families.

## Figures and Tables

**Table 1:** Demographic characteristics of the study participants.

Participant number	Age (year)	Gender	Type of relation	Caregiving history (year)
P1	27	Female	Wife	3
P2	44	Male	Husband	4
P3	32	Female	Wife	2
P4	54	Male	Husband	7
P5	35	Female	Wife	4
P6	29	Female	Wife	3
P7	48	Female	Mother	4
P8	36	Female	Wife	3
P9	48	Female	Mother	2
P10	50	Male	Husband	6
P11	30	Female	Wife	2
P12	47	Female	Mother	4
P13	49	Female	Mother	3
P14	38	Female	Wife	2
P15	26	Female	daughter	4
P16	46	Male	Husband	3
P17	38	Female	Wife	4
P18	48	Female	Mother	3

**Table 2:** Categories and subcategories related to dealing with present situation.

Main categories	Subcategories
Using spirituality	*Trust in God*
	*Sincere worship*
	*Faith in God*
	*Belief in destiny*
	*Relying on religious values*

Living with hope	*Hopefulness*
	*Optimism*

Experiencing persistence and stability	*Maintaining morale*
	*Self-reliance*
	*Independence in care*
	*Managing the conditions*

Seeking support	*Seeking financial support*
	*Seeking social support*
	*Seeking emotional support*

Seeking alternative treatments	*Discontinuing current treatment*
	*Seeking more effective medications*
	*Testing new medication*
	*Resort to traditional remedy and nondrug treatment*

## Additional Points

*Study Limitations. *The small sample within a particular cultural setting could have limited our findings, especially in a country where MS remains unknown to the public and widely misunderstood. Cultural understandings of MS as a disability were limiting for the result transferability beyond its immediate context. However this limitation is also a key feature of this paper as there are relatively few published studies exploring MS in Iran and examining how patients and families cope with MS, because studies from the western countries may not be easily transferable to the Iranian culture.

## References

[1] Murray T. (2005). *Multiple sclerosis: the history of a disease*.

[2] Fauci A., Braunwald E., Kasper D., Hauser S., Longo D., Jameson J. (2008). *Harrison's Principles of Internal Medicine*.

[3] Brass S. D., Li C.-S., Auerbach S. (2014). The underdiagnosis of sleep disorders in patients with multiple sclerosis. *Journal of Clinical Sleep Medicine*.

[4] Bendszus M., Storch-Hagenlocher B. (2013). Multiple sclerosis and other demyelinating diseases. *Inflammatory Diseases of the Brain*.

[5] Buhse M. (2008). Assessment of caregiver burden in families of persons with multiple sclerosis. *Journal of Neuroscience Nursing*.

[6] Strickland K., Worth A., Kennedy C. (2015). The experiences of support persons of people newly diagnosed with multiple sclerosis: An interpretative phenomenological study. *Journal of Advanced Nursing*.

[7] Uccelli M. M. E. (2014). The impact of multiple sclerosis on family members: a review of the literature. *Neurodegenerative Disease Management*.

[8] MS International Federation: Epidemiology of MS. http://www.msif.org/research/epidemiology-of-ms/.

[9] Ghafari S., Khoshknab M. F., Norouzi K., Mohamadi E. (2014). Spousal support as experienced by people with multiple sclerosis: a qualitative study. *Journal of Neuroscience Nursing*.

[10] Nabavi S. 12th International Congress of Multiple Sclerosis. http://www.ghatreh.com/news/nn27862825.

[11] Dennison L., Moss-Morris R., Chalder T. (2009). A review of psychological correlates of adjustment in patients with multiple sclerosis. *Clinical Psychology Review*.

[12] Pavlou M., Johnson P., Davis F. A., Lefebvre K. (1979). A program of psychologic service delivery in a multiple sclerosis center. *Professional Psychology: Research and Practice*.

[13] Pakenham K. I. (2001). Application of a stress and coping model to caregiving in multiple sclerosis. *Psychology, Health and Medicine*.

[14] Ehrensperger M. M., Grether A., Romer G. (2008). Neuropsychological dysfunction, depression, physical disability, and coping processes in families with a parent affected by multiple sclerosis. *Multiple Sclerosis*.

[15] McCabe M. P., O'Connor E. J. (2012). Why are some people with neurological illness more resilient than others?. *Psychology, Health and Medicine*.

[16] Arnett P. A. (2007). Caregiver burden in multiple sclerosis. *Journal of Neurology, Neurosurgery and Psychiatry*.

[17] (2000). Agency for healthcare research and quality: the characteristics of long-term care users. *AHRQ Research Report*.

[18] Glozman J. M. (2004). Quality of life of caregivers. *Neuropsychology Review*.

[19] McCabe M. P., McKern S., McDonald E. (2004). Coping and psychological adjustment among people with multiple sclerosis. *Journal of Psychosomatic Research*.

[20] Carton H., Loos R., Pacolet J., Versieck K., Vlietinck R. (2000). A quantitative study of unpaid caregiving in multiple sclerosis. *Multiple Sclerosis*.

[21] De Judicibus M. A., McCabe M. P. (2007). The impact of the financial costs of multiple sclerosis on quality of life. *International Journal of Behavioral Medicine*.

[22] Pakenham K. I. (2007). The nature of caregiving in multiple sclerosis: Development of the caregiving tasks in multiple sclerosis scale. *Multiple Sclerosis*.

[23] Jennum P., Wanscher B., Frederiksen J., Kjellberg J. (2012). The socioeconomic consequences of multiple sclerosis: a controlled national study. *European Neuropsychopharmacology*.

[24] Rotstein Z., Hazan R., Barak Y., Achiron A. (2006). Perspectives in multiple sclerosis health care: Special focus on the costs of multiple sclerosis. *Autoimmunity Reviews*.

[25] Ytterberg C., Johansson S., Gottberg K., Holmqvist L., von Koch L. (2008). Perceived needs and satisfaction with care in people with multiple sclerosis: A two-year prospective study. *BMC Neurology*.

[26] Abolhassani S., Yazdannik A., Taleghani F., Zamani A. (2015). Expectations of multiple sclerosis patients and their families: A qualitative study in Iran. *Iranian Red Crescent Medical Journal*.

[27] Graneheim U. H., Lundman B. (2004). Qualitative content analysis in nursing research: concepts, procedures and measures to achieve trustworthiness. *Nurse Education Today*.

[28] Elo S., Kyngäs H. (2008). The qualitative content analysis process. *Journal of Advanced Nursing*.

[29] Godau R. (2004). Qualitative data analysis software: MAXqda and MAXdictio. *Qualitative Research Journal*.

[30] Streubert H. J., Carpenter D. R. (2011). *Qualitative Research in Nursing: Advancing The Humanistic Imperative*.

[31] Betz C. L., Hunsberger M., Wright S. (1994). *Family-centered nursing care of children*.

[32] Otong D. (1995). *Psychiatric nursing: Biological and behavioral concepts*.

[33] Paterson B. (2001). Myth of empowerment in chronic illness. *Journal of Advanced Nursing*.

[34] Lazarus R., Folkman S. (1984). *Stress, Appraisal, and Coping*.

[35] Dadkhah B., Valizadeh S., Mohammadi E., Hassankhani H. (2014). The perception of trauma patients from social support in adjustment to lower-limb amputation: a qualitative study. *Indian Journal of Palliative Care*.

[36] Hassankhani H., Taleghani F., Mills J., Birks M., Francis K., Ahmadi F. (2010). Being hopeful and continuing to move ahead: religious coping in Iranian chemical warfare poisoned veterans, a qualitative study. *Journal of Religion and Health*.

[37] Taleghani F., Yekta Z. P., Nasrabadi A. N., Käppeli S. (2008). Adjustment process in Iranian women with breast cancer. *Cancer Nursing*.

[38] Whittemore R., Dixon J. (2008). Chronic illness: The process of integration. *Journal of Clinical Nursing*.

[39] Soderberg J. (1992). *MS and The Family System, Multiple Sclerosis and The Family*.

[40] Borneman T., Stahl C., Ferrell B. R., Smith D. (2002). The concept of hope in family caregivers of cancer patients at home. *Journal of Hospice and Palliative Nursing*.

[41] Rahmani A. (2012). *The process of hopefulness in patients with cancer: A grounded theory study [Ph.D. thesis]*.

[42] Bull M. J. (2014). Strategies for sustaining self used by family caregivers for older adults with dementia. *Journal of Holistic Nursing*.

[43] Keefe F. J., Ahles T. A., Porter L. S. (2003). The self-efficacy of family caregivers for helping cancer patients manage pain at end-of-life. *Pain*.

[44] Reynolds F., Prior S. (2003). "Sticking jewels in your life": Exploring women's strategies for negotiating an acceptable quality of life with multiple sclerosis. *Qualitative Health Research*.

[45] Pakenham K. I., Bursnall S. (2006). Relations between social support, appraisal and coping and both positive and negative outcomes for children of a parent with multiple sclerosis and comparisons with children of healthy parents. *Clinical Rehabilitation*.

[46] Thielemann P. A., Conner N. E. (2009). Social support as a mediator of depression in caregivers of patients with end-stage disease. *Journal of Hospice and Palliative Nursing*.

[47] Clark A. M., Barbour R. S., McIntyre P. D. (2002). Preparing for change in the secondary prevention of coronary heart disease: A qualitative evaluation of cardiac rehabilitation within a region of Scotland. *Journal of Advanced Nursing*.

[48] Rosland A.-M., Heisler M., Janevic M. R. (2013). Current and potential support for chronic disease management in the United States: The perspective of family and friends of chronically ill adults. *Families, Systems and Health*.

[49] Aymerich M., Guillamón I., Jovell A. J. (2009). Health-related quality of life assessment in people with multiple sclerosis and their family caregivers. A multicenter study in Catalonia (Southern Europe). *Patient Preference and Adherence*.

[50] Hassankhani H. (2009). *Process of Life with Chemical Injury of The Veterans*.

[51] Devine D., Parker P. A., Fouladi R. T., Cohen L. (2003). The association between social support, intrusive thoughts, avoidance, and adjustment following an experimental cancer treatment. *Psycho-Oncology*.

